# Risk analysis for occurrences of schistosomiasis in the coastal area of Porto de Galinhas, Pernambuco, Brazil

**DOI:** 10.1186/1471-2334-14-101

**Published:** 2014-02-23

**Authors:** Elainne Christine de Souza Gomes, Onicio Batista Leal-Neto, Fernando José Moreira de Oliveira, Julyana Viegas Campos, Reinaldo Souza-Santos, Constança Simões Barbosa

**Affiliations:** 1 Laboratory of Parasitology, Vitoria Academic Center, Federal University of Pernambuco, Recife, Pernambuco, Brazil; 2 Schistosomiasis Laboratory and Reference Service, Department of Parasitology, Aggeu Magalhães Research Center, Fiocruz, Recife, Pernambuco, Brazil; 3 Department of Public Health, Aggeu Magalhães Research Center, Fiocruz, Recife, Pernambuco, Brazil; 4 Departmant of Endemic Diseases, National School of Public Health, Fiocruz, Rio de Janeiro, Brazil

**Keywords:** Schistosomiasis, Risk analysis, Spatial analysis, Pernambuco, Brazil

## Abstract

**Background:**

Manson’s schistosomiasis continues to be a severe public health problem in Brazil, where thousands of people live under the risk of contracting this parasitosis. In the Northeast of Brazil, schistosomiasis has expanded from rural areas to the coast of Pernambuco State, where the intermediate host is *Biomphalaria glabrata* snails. This study aims at presenting situational analyses on schistosomiasis at the coastal locality of Porto de Galinhas, Pernambuco, Brazil, by determining the risk factors relating to its occurrence from the epidemiological and spatial perspectives.

**Methods:**

In order to gather prevalence data, a parasitological census surveys were conducted in 2010 in the light of the Kato-Katz technique. Furthermore, malacological surveys were also conducted in the same years so as to define the density and infection rates of the intermediate host. Lastly, socioeconomic-behavioral survey was also conducted to determine the odds ratio for infection by *Schistosoma mansoni*. Based on these data, spatial analyses were done, resulting in maps of the risk of disease transmission. To predict the risk of schistosomiasis occurrence, a multivariate logistic regression was performed using R 2.13 software.

**Results:**

Based on prevalence, malacological and socioeconomic-behavioural surveys, it was identified a prevalence of 15.7% in the investigated population (2,757 individuals). Due to the malacological survey, 36 breeding sites were identified, of which 11 were classified as foci of schistosomiasis transmission since they pointed out snails which were infected by *Schistosoma mansoni.* Overall, 11,012 snails (*Biomphalaria glabrata*) were collected. The multivariate regression model identified six explanatory variables of environmental, socioeconomic and demographic nature. Spatial sweep analysis by means of the Bernoulli method identified one statistically significant cluster in Salinas (RR = 2.2; p-value < 0.000), the district with the highest occurrence of cases.

**Conclusions:**

Based on the resulting information from this study, the epidemiological dimensions of this disease are significant and severe, within the scenario of schistosomiasis in Pernambuco state. The risk factors which were identified in the predictive model made it clear that the environmental and social conditions influence on the schistosomiasis occurrences.

## Background

Occurrences of schistosomiasis, which is an endemic disease in several countries, are modulated not only by the biological components of the snail that is its vector and definitive host of *Schistosoma mansoni* but also by complex social and cultural processes related to human behaviour and disorderly occupation of urban spaces [1-3]. It has been perceived the importance of socioeconomic characteristics and human behaviour regarding water contact and sanitation condition, as these factors are directly related to the frequency and intensity of human exposure to transmission foci of this disease [4]. The degree of relationship between these factors could determine the risk and transmission rates in localities where the disease became established [5].

The study of patterns of contact with the use of water at breeding sites for *Biomphalaria* on the coast of the state of Pernambuco showed that the rainy season conditioned contact and involuntary exposure to that vector, insofar as 92% of the surveyed individuals said that they had not had any contact with contaminated water during the dry season, whilst 46% also reported that they had been in contact with natural breeding sites when they overflowed at the time of the rains, causing exposure and contamination inside their own homes [6]. Lack of sanitation was highlighted as a socioeconomic determinant for schistosomiasis, inasmuch as this allowed faecal contamination of water accumulations and promoted the beginning of the disease transmission cycle [7]. On the periphery of urban localities, organic sediments originated by used water and open-air sewage not only provided a source of nourishment to the molluscs but also maintained the peridomestic foci of vectors and ensured schistosomiasis transmission [8].

The socioeconomic profile was also indicated as a determinant for occurrences of the disease. Low schooling levels among the heads of families, the amount of people per habitation as well as poor financial conditions among those families are considered to be risk factors to incur schistosomiasis [9,10]. The conjoint analysis of these factors could be able to establish a risk value that could also be expressed as a “risk ratio” by means of regression analysis [7,9]. In this manner, an attempt can be made to understand the schistosomiasis transmission process in an ecological and dynamic way.

Within this perspective, spatial analysis on health events has proved to be a valuable tool for epidemiological studies, as it enables information to be aggregated, health indicators to be established, landscapes to be characterized, and categorizing schistosomiasis occurrences to be modulated. Details of the environmental conditions that interfere in the population’s health can be illustrated on maps that present the spatial distribution of areas at risk, thus it makes it possible for epidemiologists to understand the dynamics of the disease, along with its variations in space and time. For spatial analysis on socioeconomic, behavioural, biological and environmental risk factors, physical and topographic maps of the studied localities, in digital format, need to be used [11]. These should contain geographical features such as: blocks, plots, constructions, water accumulations, relief and vegetation, and they should be used jointly with geodesic coordinates which could be provided by the global positioning system (GPS) and spatial statistical software [12].

Spatial statistical analysis has been used as a tool for predicting occurrences of schistosomiasis in studies conducted in the Philippines and the Caribbean [13,14] and widely used in studies on this health hazard as it has produced inferences regarding associations between infection and environmental variables. Field studies and modelling have improved the understanding of the spatial epidemiology of schistosomiasis in Africa, and have shown its relevance in planning schistosomiasis control programmes [5,15].

Data on points of occurrence of an event can be analysed based on identifying spatial agglomerations known as ‘clusters’. Some of those clusters analyses provide geostatistical parameters, such as the relative risk for some areas identified by the cluster and p-value as it is illustrated in Bernoulli’s method [16]. This technique has been used in several fields of studies to detect the spatial risk of disease occurrence [16-19]. Areas at risk can also be estimated empirically, based on knowledge of the territorial extent, by creating ‘buffers’ which are consisted of risk bands, from identified areas of influence for those variables. This type of analysis enables better spatial understanding of the transmission and occurrence of a disease.

Concerning schistosomiasis, Brazil is considered the most affected country in the Americas. It has been estimated that around 30 million people are exposed to the risk of contracting this disease [20,21], and its prevalence may have already surpassed eight million individuals [22]. Moreover, for over a decade, schistosomiasis has been ceasing to be a characteristically rural endemic disease and has been expanding into urban and coastal areas of Brazil, and by such it has been exposing a new portion of the population to the risk of becoming infected [1,2,23-27].

Within this perspective, the present study aims at presenting the results from a situational analysis on schistosomiasis and determining the extent of the epidemiological and spatial risks of this disease at the coastal locality of Porto de Galinhas, Pernambuco, Brazil.

## Methods

### Study area

**Figure 1:**
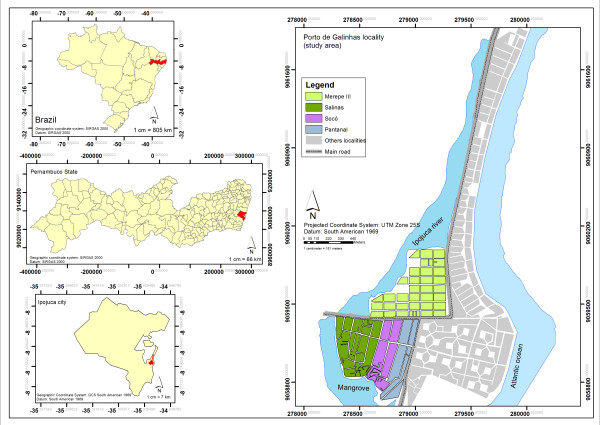
**Map of Porto de Galinhas, Ipojuca, Pernambuco state - Brazil.** Fonte: Gomes ECS *et al.*, 2011 [29].

This study was developed at the locality of Porto de Galinhas, in the municipality of Ipojuca, on the southern coast of the state of Pernambuco, at a distance of 60 km from the state capital city, Recife (Figure 1). This locality was chosen in view of records of breeding sites for *B. glabrata* and a high number of cases that have been registered over the last decade [28]. For the malacological survey and schistosomiasis investigation, the entire area of Porto de Galinhas was taken into consideration (videlicet: Merepe I, II and III; Salinas, Socó, Pantanal and Vila de Porto).

### Data gathering and analysis

#### Mapping of the locality

Georeferenced mapping of the locality was performed in May and June 2010, using a GPS receiver (Garmin, model Vista Cx) configured in the UTM (Universal Transverse Mercator) projection system with the SAD 69 datum. The mapping included streets, main buildings and water accumulations containing the vector mollusc, and the latter were subsequently classified as breeding sites or foci of schistosomiasis transmission. After the human prevalence survey on schistosomiasis had been taken, all the residences in which at least one individual had undergone a parasitological faeces examination (with both positive and negative results for schistosomiasis) were also georeferenced.

For all the spatial analyses on the data, only the localities of Merepe III, Salinas, Socó and Pantanal were taken into consideration. These were the areas in which more than 70% of the permanent population of Porto de Galinhas were living, as it was informed by the Municipal Health Department (Figure 1).

#### Malacological survey

Snails were collected at demarcated stations at the breeding sites that had been identified, in random sampling every month over a one-year period (from July 2010 to June 2011). In each station the snails were caught by using scoops and tweezers during every15-minute periods, and stored properly in moistened and ventilated plastic jars, which were also labelled according to the number of the corresponding breeding site. Then, they were taken to the Schistosomiasis Laboratory and Reference Service at the Aggeu Magalhães Research Center – Fiocruz, in order to be examined. To diagnose infection and identify transmission foci (i.e. breeding sites in which snails infected with *S. mansoni* were found), the snails were exposed to artificial light so that the cercariae would be expelled [30]. Snails that were found to be negative were re-examined using the same technique 15 days after the first examination. Those that continued to be negative were then crushed, in groups of 12, on glass plates whose dimensions were 15 × 9 cm, and then they were examined individually under a stereoscopic microscope in search of sporocysts (larval stages) of *S. mansoni*[31]. The snail density per breeding site was defined according to the absolute number of snails collected in each breeding site per month. To determine the natural infection rate or infectivity rate, it was calculated the proportion of the molluscs that were positive for *S. mansoni* in relation to the total number of molluscs examined. In this manner, the monthly and annual infection rates were calculated for each breeding site and transmission focus, in order to estimate the potential for infectivity and disease transmission at each focus.

#### Prevalence survey

Between August and December 2010, a coproscopic census survey was conducted based on voluntary registration and participation of individuals living in Porto de Galinhas. After signing the consent form provided by the ethics committee from Aggeu Magalhães Research Center – who approved the whole study, it was given to them a plastic container to collect stool sample, which could be identified by the name of each participant that agreed in taking part of this research. Thus, stool samples were collected and sent to the laboratory in order to be examined.

To define the number of cases of schistosomiasis and the parasite load in these individuals, parasitological examinations were conducted on their faeces by means of the Kato-Katz method [32], with one sample from each patient, from which two slides were examined. The prevalence of schistosomiasis in Porto de Galinhas was defined from the results of the parasitological faeces examinations, using the following formula: total number of positive individuals/ total number of individuals examined × 100. The human infection rate or parasite load of each individual was defined by multiplying the number of eggs found on each slide by the constant 24, in order to obtain the number of eggs per gram of faeces (EPG). Since two slides were made from each sample, the result related to the parasite load for each individual was formed by the arithmetic mean of the EPG from the two slides examined.

#### Risk analysis

In March 2011, sanitary, environmental, socioeconomic and behavioural data were gathered by means of a questionnaire which was applied to all the individuals who participated in the study and were available to answer the questions. The demographic data related to age and sex were obtained from the coproscopic survey forms that had been filled out by the time of registering of these individuals.

With the aim of creating a model that would enable predictions of the risk of schistosomiasis occurrence, multivariate logistic regression was performed in *R* software. The dependent variable was taken to be the occurrence of the case (0 = non-case; 1 = case), and the independent or explanatory variables were taken to be the environmental, social, economic and behavioural data that had been gathered by means of the socioenvironmental questionnaire, along with the demographic data of age (age group) and sex. These independent variables were categorized as zero for protection factors and one for risk factors, with the exception of age, which risk factor was stratified as 1, 2 or 3. Thus, the following categories were set up: home with piped water (0), home without piped water (1); home in which no water was accumulated in the backyard in winter (0), home with accumulated water in the backyard in winter (1); home in which no water was accumulated in the backyard in summer (0), home with accumulated water in the backyard in summer (1); sewage disposal into the ‘general sewage system’ (0), sewage disposal into ‘septic pit or open ditch’ (1); schooling level of the head of the family: ‘high school or university level’ (0), ‘illiterate or elementary education level’ (1); age group: ‘0 - 9 years old or > 60 years old’ (0), ‘10 - 19 years old’ (1), ‘20 - 39 years old’ (2), ‘40 - 60 years old’ (3); number of members of the family per home: ‘< 5 people’ (0), ‘≥ 5 people’ (1); not stepping in water when going out from home in winter (0), stepping in water when going out from home in winter (1); not stepping in water when going out from home in summer (0), stepping in water when going out from home in summer (1); living in an asphalted street (0), not living in an asphalted street (1); family income ‘> 1 minimum monthly salary’ (0), ‘≤ 1 minimum monthly salary’ (1); sex ‘female’ (0), ‘male’ (1); length of time living at the locality ‘< 1 year’ (0), ‘> 1 year’ (1).

Univariate models were run with the dependent variable and each of the independent variables, and the regressions that presented p-values less than or equal to 0.25 were selected for the multivariate model. After forming the multivariate logistic model, the stepwise forward model was applied and the variables that presented p-values less than 0.05 were accepted to make up the final model. The chosen model was justified as the model that presented the lowest value for the Akaike Information Criterion (AIC) and the equation that best explained the problem.

#### Spatial risk

To establish the potential risk that a breeding site for *B. glabrata* would become a focus for schistosomiasis transmission, a thematic map demonstrating the occurrences of these breeding sites and foci was made in the form of kernel map of parasite load in human cases per home (over a radius of 200 meters [33]). Based on the distribution of the foci, a multiple ring buffer map was compiled, with pre-established distances of 50 meters (radius of a block), in order to estimate the expansion of the foci during rainy periods. Both analyses were performed using the *ArcGis 10* software – Spatial Analysis Extension*.*

A sweep analysis was also performed to identify areas at greater risk of occurrences of cases (clusters), by using the geolocation of cases and non-cases. For this, the *SaTScan 9.1.1* software was a useful tool and Bernoulli’s method was used as well, such that the variables of “case” and “non-case” were dichotomous [18]. Based on that method, it is possible to identify clusters containing geostatistical representations, such as the relative risk, radius of the area at risk, and p-value. The clusters were obtained and the risk was determined through comparing the “number of observed cases” with the “number of expected cases”. The significance test was based on the likelihood test, in which the p-value was obtained by means of the Monte Carlo test. Only the clusters with p-values < 0.05 were considered in the results. Based on the results from this analysis, maps presenting areas at greater risk of occurrences of cases were designed, and these were correlated with the socioenvironmental information that had been gathered previously and were overlain with a satellite image of the area, by means of the *ArcGis 10* software - Spatial Analysis Extension*.* These images were generated through panchromatic (PAN) and multispectral (MS) sensors on board of the QuickBird satellite, with spatial resolution of 0.6 (August, 2006) and 0.5 meters (June, 2010).

## Results

**Figure 2:**
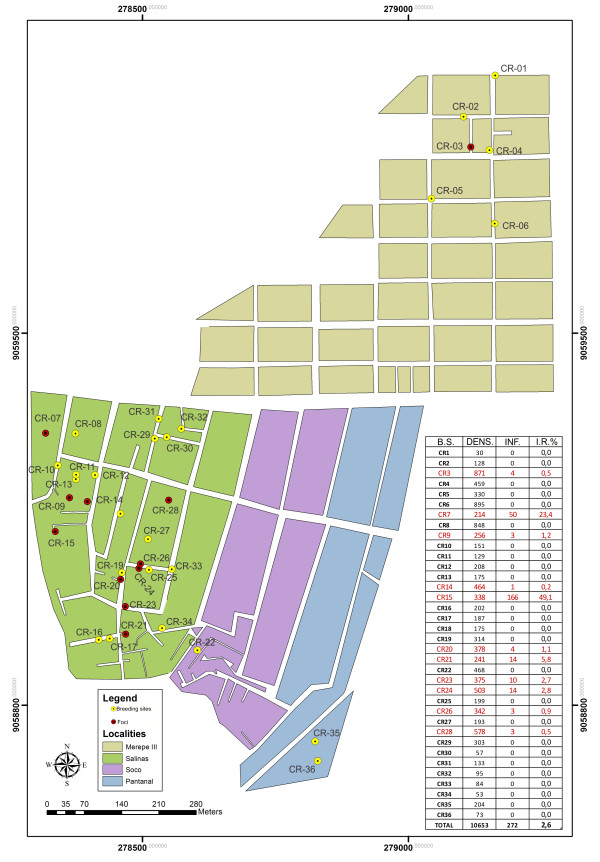
**Spatial distribution of breeding sites and foci of *B. glabrata *** **in Porto de Galinhas, Ipojuca, Pernambuco - Brazil.**

Over the course of one year of malacological surveys, 11,012 snails of the species *B. glabrata* were collected from 36 breeding sites (I.R. 2.6%) that were identified at the studied locality. Of these, 11 sites (representing 30.5% of the total breeding sites) were classified as foci of schistosomiasis transmission because they presented snails infected with *S. mansoni*. Thus, there were 272 positive snails in total. The spatial distribution of the breeding sites and schistosomiasis foci per district within the studied area, along with the respective population densities and infection rates of the snails, can be seen in Figure 2. Merepe III presented six breeding sites, of which one was a focus of transmission; Salinas was the district with the largest numbers of breeding sites and foci: 27 and 10, respectively; Socó and Pantanal only had three breeding sites between them. The foci with the highest snail infection rates were breeding site 7 (23.4%) and 15 (49.1%), which were the closest sites to the flooded mangrove swamp area. It was also observed that, although the infection rates were mostly low, there were high densities of snails at the foci and a large number of snails were collected over the complete year of investigation.

For the epidemiological census survey, 5,607 individuals were registered. Of these, 2,757 adhered to the investigation by returning with the biological samples (faeces) for the parasitological diagnoses. Of these, 434 were positive for *S. mansoni.* The prevalence of schistosomiasis in Porto de Galinhas was 15.7%. The district with the most significant number of cases was Salinas, followed by Sóco and Pantanal. Merepe III and Vila de Porto that were the localities with least occurrence of cases, and as such, they presented the lowest prevalence rates (Table 1). The localities Merepe I and II are not represented in this table because their populations’ adherence to the survey was low, insomuch as only 57 individuals were examined, of whom nine were cases of the disease.

**Table 1:** Occurrences of schistosomiasis cases and prevalence according to district, Porto de Galinhas, Ipojuca, Pernambuco, 2010

**Districts**	**Sampled (*n*)**	**Positives**			**95% CI**
		**No**	**%**	**Prevalence (%)**	
Merepe III	315	14	3.2	4.4	1.8 – 5.5
Salinas*	1263	259	59.7	20.6*	54.9 – 64.3
Socó*	590	96	22.1	16.3*	18.4 – 26.4
Pantanal*	291	40	9.2	13.7*	6.7 – 12.4
Vila de Porto	241	16	3.7	6.6	2.2 – 6.0
Total	2700	425	97.9	15.7	–

*Frequency variation statistically significant (CI).

To determine the risk factors relating to occurrences of schistosomiasis at the study localities, 549 questionnaires were applied at the homes that formed part of the sample, which represented 60.3% of the totality. These questionnaires delineated the socioeconomic, behavioural and sanitary-environmental profile of 1,719 individuals (62.3% of the total population samples) representing 1,429 “non-cases” (61.5% of the non-cases) and 290 ‘cases’ (66.8% of the cases). With the objective of identifying the risk factors for occurrences of schistosomiasis, all the variables presented in Table 2 were included in a logistic regression analysis, which identified the eight explanatory variables shown in Table 3.

**Table 2:** Socioeconomic, behavioural and environmental characteristics of the population sampled in Porto de Galinhas Ipojuca, Pernambuco 2010

**Independent variables**	**Cases**			**Non-cases**		
	**No**	**%**	**95% CI**	**No**	**%**	**95% CI**
With piped water	286	98.6	96.5 – 99.6	1.381	96.6	95.5 – 97.5
Without piped water	4	1.4	0.4 – 3.5	48	3.4	2.5 – 4.5
No accumulation of water in backyard in winter	89	30.7	25.4 – 36.3	591	41.4	38.8 – 44.0
With accumulated water in backyard in winter	201	69.3	63.7 – 74.6	838	58.6	56.0 – 61.2
No accumulation of water in backyard in summer	206	71.0	65.4 – 76.2	1.046	73.2	70.8 – 75.5
With accumulated water in backyard in summer	84	29.0	23.8 – 34.6	383	26.8	24.5 – 29.2
Sewage disposal: general system	6	2.1	0.8 – 4.4	51	3.6	2.7 – 4.7
Sewage disposal: pit or open ditch	284	97.9	95.6 – 99.2	1.378	96.4	95.3 – 97.3
Head of family’s schooling: high school or university	36	12.7	9.1 – 17.2	321	22.9	20.7 – 25.2
Head of family’s schooling: illiterate or elementary school	247	87.3	82.8 – 90.9	1.083	77.1	74.8 – 79.3
Age group: 0–9 and > 60 years	54	12.6	9.7 – 16.3	682	29.4	27.6 – 31.3
Age group: 10–19 years	99	23.2	19.3 – 27.5	384	16.6	15.1 – 18.2
Age group: 20–39 years	196	45.9	41.1 – 50.8	765	33.0	31.1 – 35.0
Age group: 40–59 years	78	18.3	14.8 – 22.3	487	21.0	19.4 – 22.7
No. of family members in home: < 5 peoples	164	56.6	50.6 – 62.3	886	62.0	59.4 – 64.5
No. of family members in home: > 5 people	126	43.4	37.7 – 49.4	543	38.0	35.5 – 40.6
Not stepping in water when going out from home in winter	34	11.7	8.3 – 16.0	298	20.9	18.8 – 23.1
Stepping in water when going out from home in winter	256	88.3	84.0 – 91.7	1.131	79.1	76.9 – 81.2
Not stepping in water when going out from home in summer	223	76.9	71.6 – 81.6	1.132	79.2	77.0 – 81.3
Stepping in water when going out from home in summer	67	23.1	18.4 – 28.4	297	20.8	18.7 – 23.0
Living in asphalted street	61	21.0	16.5 – 26.2	480	33.6	31.2 – 36.1
Not living in asphalted street	229	79.0	73.8 – 83.5	949	66.4	63.9 – 68.8
Family income: > 1 minimum monthly salary	208	71.7	66.2 – 76.8	1.166	81.6	79.5 – 83.6
Family income: < 1 minimum monthly salary	82	28.3	23.2 – 33.8	263	18.4	16.4 – 20.5
Female sex	175	40.3	35.7 – 45.1	1.270	54.7	52.6 – 56.7
Male sex	259	59.7	54.9 – 64.3	1.053	45.3	43.3 – 47.4
Length of time living at locality: < 1 year	5	1.7	0.6 – 4.0	58	4.1	3.1 – 5.3
Length of time living at locality: > 1 year	285	98.3	96.0 – 99.4	1.371	95.9	94.7 – 96.9

**Table 3:** Results of multivariate logistic regression

**Explanatory variables**	**OR**	**95% CI**	**p-value**
With accumulated water in backyard in winter	1.40	1.06 – 1.88	0.020
Head of family’s schooling: illiterate or elementary school	1.62	1.11 – 2.42	0.015
Age group: 10–19 years	2.35	1.53 – 3.65	0.000
Age group: 20–39 years	2.89	1.98 – 4.28	0.000
Age group: 40–59 years	2.02	1.30 – 3.16	0.002
Not living in asphalted street	1.57	1.14 – 2.17	0.006
Family income: < 1 minimum monthly salary	1.46	1.07 – 1.98	0.017
Male sex	1.71	1.31 – 2.24	0.000

AIC 1441.9.

The first explanatory variable for occurrences of schistosomiasis cases in Porto de Galinhas was ‘the accumulation of water in the backyard in winter’. Table 2 shows that 70% of the cases presented this condition. According to the regression model (Table 3), these cases represented a 40% greater risk of contracting schistosomiasis in comparison with the individuals without this condition. This explanatory variable is extremely important, whereas during the rainy season breeding sites were observed not only in the streets but also in the backyards and even inside homes. ‘Low schooling level of the head of the family’ was also a risk factor for occurrences of schistosomiasis, and around 90% of the individuals who were positive for schistosomiasis presented this condition (Table 2). The individuals exposed to this condition were at 60% greater risk of acquiring the disease (Table 3).

Age group was the variable that presented the highest risk that an individual would become a ‘case’. This risk was twice as high for the age groups between 10 and 59 years, in comparison with the reference age group. However, attention was drawn to the young adult age group (20–39 years), which alone accounted for almost half of the schistosomiasis cases registered at this locality (Table 2). Thus, the individuals in this age group were at almost three times greater risk of acquiring schistosomiasis, compared with the individuals in the reference age group, as shown in the regression model.

The urban infrastructure was also considered to be a risk factor, regarding the ‘paving of the streets’. Individuals who were living in non-asphalted streets were at 57% greater chance of becoming ill, and it should be noted that almost 80% of the cases occurred at unpaved localities (Table 2). Individuals living in homes in which the family income was less than one minimum monthly salary were at 46% greater chance of becoming ill, and males were at 70% greater chance of acquiring this parasitosis, in comparison with females.

**Figure 3:**
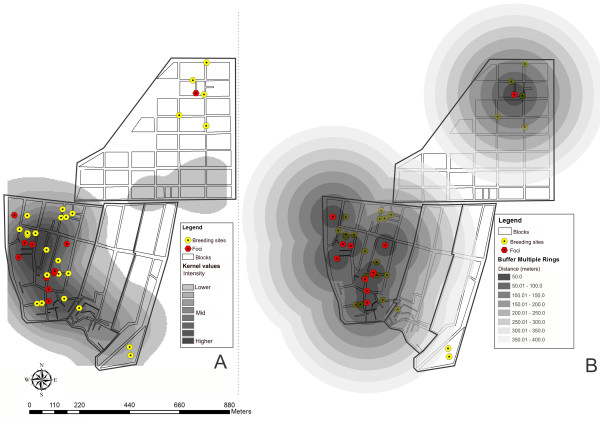
**Maps showing real and potential spatial risk of schistosomiasis transmission, Porto de Galinhas, Ipojuca, Pernambuco - Brazil. (A)** Kernel map of parasite load per household X breeding sites and foci of Biomphalaria glabrata; **(B)** Buffer map of the distance between the foci of Biomphalaria glabrata.

In addition to this regression analysis, analysis models estimating the spatial risk were generated in an attempt to better understand occurrences of schistosomiasis in this area, insomuch as that the environment is an integral and non-dissociable part of the transmission method for this disease. Over the one-year malacological data-gathering period, it was observed that the rainy period directly influenced on the occurrence, density and rate of snail infection. Therefore, it was sought to determine the extent in which this variable influenced the distribution of the breeding sites for *B. glabrata* and the foci of schistosomiasis transmission. Figure 3 presents a kernel map for the mean parasite load among the individuals who were positive for schistosomiasis per home. On this map, the distribution of breeding sites and transmission foci can be seen. Most of them, except for those located in Merepe III, were within the risk area that presented the parasite load of the cases. This means that although no snails that were positive for *S. mansoni* were found at the breeding sites, these sites were at risk of becoming schistosomiasis transmission foci at any time.

The potential risk of schistosomiasis transmission can be seen in Figure 3(A). This shows that the majority of the breeding sites were in areas in which environmental contamination by the faeces of parasitized individuals existed. The coexistence of foci of transmission in the same region corroborates this observation. One way of better comprehending this potential risk and the influence of the rainfall regime on schistosomiasis transmission can be seen in Figure 3(B). This map provides an estimate of the intensity of exposure of the breeding sites to the risk related to the expansion of the foci, caused by the rainy season in the region. Even if the breeding sites were not originally in contact with environments contaminated by parasitized individuals, they would be vulnerable to the potential risk of becoming foci during rainy periods. This map grades the severity of the risk in 50-meter bands, and it also covers the entire region that becomes flooded during the rainy season. It was observed that at some places the rings converged, thereby indicating that the potential risk was even greater there. So these breeding sites were under the influence of areas of expansion from several transmission foci, i.e. under the influence of several contaminated areas.

**Figure 4:**
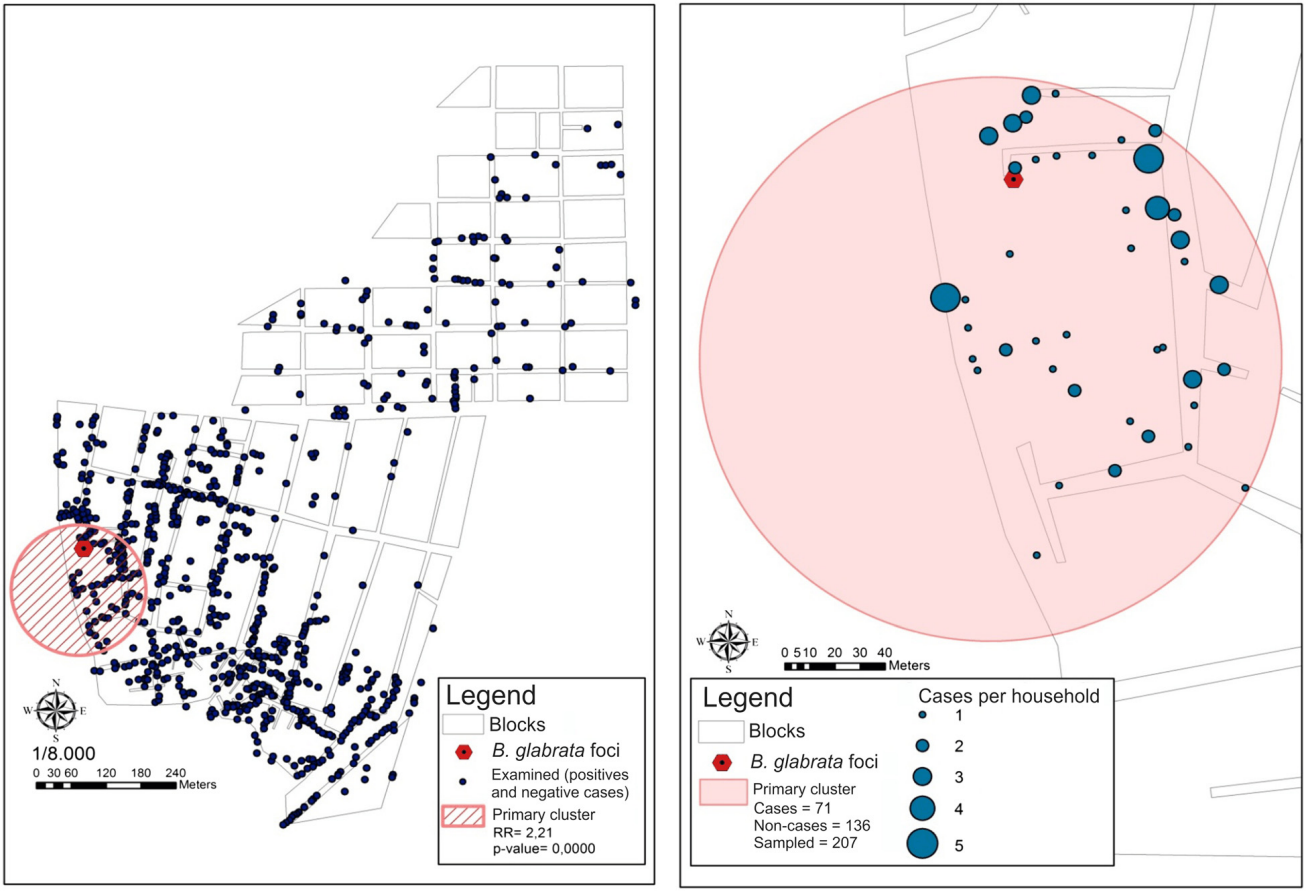
Maps showing spatial sweep analysis on schistosomiasis cases in 2010, Porto de Galinhas, Ipojuca, Pernambuco - Brazil (Bernoulli’s method – SaTScan software).

**Figure 5:**
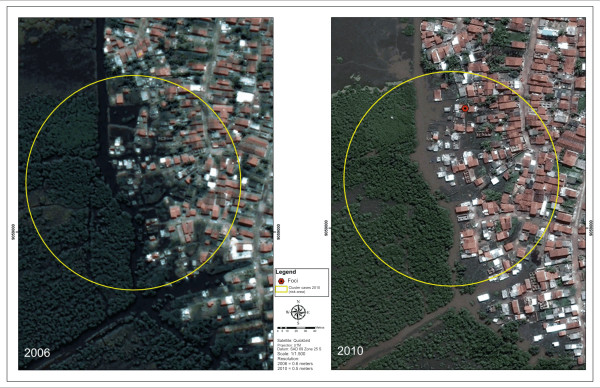
Environmental changes in the identified area of risk in the sweep analysis on schistosomiasis cases 2010, Porto de Galinhas, Ipojuca – Brazil.

The results from the sweep analysis using Bernoulli’s method identified 37 clusters, but only one was statistically significant (Figure 4A). This cluster was in Salinas and presented a radius of influence of 117 meters, thus encompassing a total of 207 sampled individuals. Of these, 34.3% were positive (71 cases) and 65.7% were negative (136 non-cases) for schistosomiasis. The individuals living in the area covered by this cluster were at 2.2 times greater risk of acquiring schistosomiasis (RR = 2.21; p-value < 0.000; 95% CI). The transmission focus with the highest snail infection rate (breeding site 15; 49.1%) recorded in this study was within the radius of this cluster (Figure 2). The information obtained from the sweep analysis on the cases made it possible to draw up a final map (Figure 5) that presented the radius of influence of the clusters on the satellite images obtained in 2006 and 2010. This map aimed to present the risk scenario for occurrences of schistosomiasis in relation to the process of disorderly urban development. It clearly illustrated the expansion of the built-up areas into the mangrove swamp areas.

It was also observed that the largest focus of transmission registered in 2010 was practically inside the mangrove swamp. This demonstrates that the process of human occupation of land is being accompanied by the expansion of the distribution of foci and breeding sites for *Biomphalaria.* Figure 5(B) shows the environmental changes that have been occurring in a rapid and uncontrolled manner at this district, with invasion of natural areas that should have been protected.

## Discussion

The biological, environmental and sanitary data gathered, the spatial analyses performed and the testimonies from the interviews with the natives made it possible to portray the current epidemiological scenario for schistosomiasis in Porto de Galinhas. The district of Salinas, which contained most of the foci of schistosomiasis transmission, is the poorest and most heavily populated district of Porto de Galinhas. It has poor basic sanitation and environmental conditions, with sewage outflow in open ditches and unpaved streets without any rainwater drainage system. These characteristics provide ideal conditions for maintaining mollusc breeding sites. In association with these factors, proximity to the mangrove swamp region makes this region vulnerable to periodic flooding caused by overflowing of the Ipojuca river, which flows around the locality. The floods carry the faecal material (exposed on the streets) and the infected snails to other localities, and this favours the appearance of new foci of the disease and exposes residents or vacationers in other districts.

Figure 3 simulates a flood and demonstrates that the rainy period has a large influence on the dynamics of schistosomiasis transmission in Porto de Galinhas. The figure shows that Salinas is the district at the greatest risk of schistosomiasis transmission, taking into consideration the chances of contact with the foci. This can be seen from the prevalence survey, in which Salinas presented a rate of 20.6%, which was the highest rate among all the localities (Table 1). Socó and Pantanal presented the second and third highest prevalence, respectively, and although these localities did not have any confirmed transmission foci, they were suitable to the influence of expansion of the areas at risk caused by floods during the rainy periods.

Young male adults were the most affected individuals by schistosomiasis, probably because they were more exposed to the vector foci located on the streets and in the backyards of people’s homes. Their contact can be highlighted as accidental, involuntary and inevitable, insomuch as that they needed to cross through these environments in order to carry out their social and labour activities. Another reason that may explain the greatest occurrence of this parasitosis among men is the fact that men tend to seek healthcare services less often than women, even if symptoms are present, which makes it difficult to diagnose and treat the disease [33].

The interviews conducted among the population of Porto de Galinhas made it possible to identify the socioeconomic, behavioural and environmental risk factors that are predictive of the risk of schistosomiasis occurrences. Among the list of variables subjected to multivariate logistic regression, ‘accumulation of water in the backyard’ was the one that presented the lowest risk (OR = 1.40; 95% CI = 1.06 – 1.88), despite being a frequent and important environmental characteristic regarding the transmission of this disease in Porto de Galinhas. Low schooling level presented by the head of the family and low family income were risk factors for the condition of becoming ill. It is known that these factors influence directly on the family’s quality of life [7] and that they indicate poor housing, dietary and health conditions. These factors are directly related to schistosomiasis, considering that this is a disease that affects needy and neglected populations. In Porto de Galinhas, the probability for young adults to contract the disease was almost three times higher than the ones that the reference age group (children and elderly people) may have. Since the young adults were constantly moving around, they were suitable to a higher risk of contact with foci of schistosomiasis transmission.

One significant environmental variable in the predictive model for schistosomiasis occurrences was ‘absence of asphalted street’. This factor is directly related to occurrences of breeding sites for *B. glabrata*, which require earth and water. Even if snails are found in the gutters and puddles of asphalted surfaces, they do not resist the desiccation that takes place in these environments, because the asphalt makes the molluscs’ process of ‘aestivation’ impossible, and consequently impedes their survival during periods of drought. The district of Salinas, which has few paved streets, is vulnerable to the presence of breeding sites and maintenance of cases.

The district of Salinas was the only one to present a statistically significant cluster of occurrences of cases, with a radius of influence of 117 meters, in which the risk of contracting the disease would be 2.2 times higher for the individuals living there (Figure 4). The cases identified in the mangrove swamp area that had suffered the impact of invasions of natural environments and was conducive to flooding that kept the plots of land underwater (Figure 5B) can be highlighted. The population living there was exposed to a higher risk of infection. This figure particularly highlights the greatest impact of the disease on peripheral areas, as the disease affects poor individuals living in invaded areas of the natural environment, without any sanitary infrastructure. The process of expansion and maintenance of schistosomiasis is induced by the way in which people have occupied peripheral and marginalized spaces in Porto de Galinhas.

## Conclusion

Based on the resulting information of this study, the predictive model for schistosomiasis transmission in the coastal area of Porto de Galinhas can be considered for showing that the epidemiological dimensions of this disease are significant and severe, within the scenario of schistosomiasis in Pernambuco. The analysis on the parasitological aspects of the transmission cycle shows that this locality is a habitat for the vector snail which has the greatest biological potential for infection. *B. glabrata* is considered to be an efficient host, since it has high resistance to *S. mansoni* infection and is capable of releasing ten times more cercariae (the larvae that infect humans) than *B. straminea*[34]. Moreover, the identified risk factors in the predictive model make it clear that the environmental and social conditions influence on schistosomiasis occurrences. This constitutes a serious epidemiological scenario, in which the neediest population has been gathered throughout peripheral areas without infrastructure, and thereby destroying natural environments and establishing suitable conditions for maintaining the transmission cycle of this endemic disease in new areas, such as the case in Porto de Galinhas.

There is an urgent need for the public authorities to take measures to ensure that Porto de Galinhas to continue its development as an important tourist centre, by eliminating the deleterious effects of diseases such as schistosomiasis. Such diseases not only have repercussions on health and socioenvironmental organization at the locality, but also may be frustrating the expectations of vacationers and tourists who head there in order to enjoy the natural beauty that this place still offers.

## Abbreviations

B. glabrata: *Biomphalaria glabrata*; B. straminea: *Biomphalaria straminea*; EPG: Eggs per gram of faeces; CPqAM: Aggeu Magalhães Research Center.

## Competing interests

The authors declare that they have no competing interests.

## Authors’ contributions

ECSG: Conception of the project; data gathering, analysis and interpretation; writing the manuscript; and important critical review of the intellectual content. OBLN: Data gathering at the fieldwork stages and spatial analysis. FJMOJ: Methodology and statistical analysis on the data. JVC: Data gathering at the fieldwork stages. RSS: Conception of the project, statistical and spatial analysis of the data. CSB: Conception and coordination of the project, critical review of the manuscript and final approval of the version to be published. All authors read and approved the final manuscript.

## Authors’ information

ECSG: PhD Public Health and MSc in Animal Biology - Professor of parasitology at Universidade Feraral de Pernambuco – Brazil.

OBLN: Public Health Master’s student at Centro de Pesquisas Aggeu Magalhães – Fiocruz – PE – Brazil.

FJMOJ: Public Health Master’s student at Centro de Pesquisas Aggeu Magalhães – Fiocruz – PE – Brazil.

JVC: Public Health Master’s student at Universidade Federal de Pernambuco – Brazil.

RSS: PhD Public Health and MSc Zoology – Head researcher of the department of endemic diseases – ENSP – Fiocruz, RJ – Brazil.

CSB: PhD Public Health and MSc in Biological Sciences – Head researcher of the department of parasitology and Coordinator of the Schistosomiasis Laboratory – CPqAM– Fiocruz – PE – Brazil.

## Pre-publication history

The pre-publication history for this paper can be accessed here:

http://www.biomedcentral.com/1471-2334/14/101/prepub
